# Registered nurses’ perceptions of their leadership close to older adults in municipal home healthcare: a cross-sectional questionnaire study

**DOI:** 10.1186/s12912-025-03210-w

**Published:** 2025-05-17

**Authors:** Erica Lillsjö, Anna Willman, Lise-Lotte Jonasson, Karin Josefsson

**Affiliations:** 1 Department of Health Sciences, Faculty of Health, Science, and Technology, Karlstad University, Karlstad, 651 88 Sweden; 2 Department of Nursing Science, School of Health and Welfare, Jönköping University, Jönköping, Sweden; 3 Faculty for Nursing and Health Science, Nord University, Bodø, 8026 Norway

**Keywords:** Registered nurse, Leadership, Specialist education, Older adult, Municipal home healthcare, Questionnaire

## Abstract

**Background:**

Registered nurses lead the nursing care close to older adults in home healthcare. It is expected that there will be an increased need for home healthcare. In addition, more advanced care is now being performed in home healthcare, leading to increased demands for registered nurses. Therefore, the aims of this study are to explore and compare registered nurses’ perceptions of their leadership close to older adults in municipal home healthcare, as well as to correlate their perceptions with age and work experience.

**Methods:**

This study is a part of a larger web-based questionnaire survey, with a non-experimental and cross-sectional design. Descriptive and analytical statistics were used. A total of *n* = 71 registered nurses leading close to older adults participated, in seven municipalities in two geographic areas in Sweden.

**Results:**

The registered nurses perceived their ability as leaders close to older adults as high. The registered nurses had neither low or high trust in care staff’s competence. They perceived to have space and access neither in a low or high degree in their work to develop sufficient competence in leadership and having nursing responsibility on an organisational level. Registered nurses’ perceptions of their leadership differed depending on whether they had a specialist education or not; those with specialist education perceived to a higher degree that they could apply their professional experience in their work; interact with the older adult and their next of kin; assess individual needs and based on a holistic view of the older adult, create good relationships with the older adults’ next of kin.

**Conclusions:**

Registered nurses’ specialist education may strengthen their leadership in home healthcare. Further research is needed to gain new knowledge of registered nurses’ leadership in home healthcare, as well as care staff’s’ experiences of registered nurses’ leadership in municipal home healthcare.

## Background

In home healthcare, registered nurses (RNs) lead nursing care close to older adults [1], defined as RNs’ leadership in direct nursing care close to the older adult, 65 years and older, their next of kin and care staff. The RNs’ leadership close to older adults implies a wide range of challenges, such as coordinating the care for the older adult and managing communication difficulties [2]. RNs in home healthcare have described that their responsibilities are not always clearly defined [3] and that their work is not clear to others [4]. At the same time, it is expected that there will be an increased need for home healthcare [5]. Globally, people are living longer and the population of older adults is expected to increase [6]. There is also a shift internationally [7], and in Sweden [8] from inpatient care in hospitals to care performed in the municipalities. In addition, patients are discharged earlier from hospitals and are cared for longer in their homes, even with complex health needs [9, 10]. This means that more advanced care [4, 11–13] and assessments are performed in home healthcare settings [13]. Therefore, there is a need for more knowledge about RNs’ leadership in home healthcare [1, 14, 15]. Thus, it is necessary to create conditions for RNs to be able to provide safe care [1], which is a challenge for RNs in home healthcare [16].

In Sweden, 21 regions and 290 municipalities [17] have responsibility for healthcare [8]. Most of the municipalities have the responsibility for home healthcare, except healthcare provided by physicians which is the region’s responsibility [8]. Home healthcare implies, such as medical interventions, rehabilitation, habilitation and nursing performed by licensed healthcare professionals and care staff [18]. The care staff have different levels of education in care or social care [19], with delegation to perform certain tasks [18], such as administration of medications and injections [20].

RNs in home healthcare have the overall nursing responsibility [18] and cooperate with professionals, such as occupational therapists, physiotherapists, physicians, care managers and care staff [21]. The communication and collaboration between RNs and care staff is important [22] since the care staff are often the ones meeting the older adult and reporting to RNs. Therefore, RNs have to rely on care staff’s judgement [22, 23]. Due to the high number of older adults within RNs’ responsibility, RNs need to delegate tasks to care staff [20, 24], such as administration of medications, injections, and insulin, as well as wound care and catheter care [20]. However, the RNs are responsible for ensuring the tasks are performed in the correct way [25, 26]. Previous research [27] shows that RNs working in municipal care for older adults with a specialist education have a higher self-rated satisfaction with their own professional competence, than RNs without specialist education. Older age and the number of working years after nursing education are also related to a higher self-rated professional competence [27]. Despite that, many of the Swedish municipalities report a lack of specialist-educated RNs [28].

RNs’ competence includes leadership [29, 30]. Leadership is not described as synonymous with management [31, 32]. Management implies duties such as planning, budgeting, organising, staffing and controlling [31]. Northouse [32] describes leadership as a process where a leader influences a group of people to reach common goals. Leadership can be categorised into different leadership styles focusing on human relationships or task completion [33]. To be a leader has been described to depend on personality characteristics, but according to Kouzes and Posner [34] it is an ability that can be learned and developed. A literature review [35] identified factors assumed to contribute to leadership, such as age, experience and education, but the results were equivocal [35].

In nursing literature, leadership is commonly described in terms of clinical leadership, nursing leadership and healthcare leadership [36], although it is not always clear if the terms refer to formal management or informal leadership [37]. In this study, RNs’ leadership close to older adults refers to RNs’ leadership in direct nursing care close to the older adult, 65 years and older, their next of kin and care staff. RNs’ leadership close to older adults in home healthcare has been described by Claesson et al. [1] as complex, implying 10 themes: trust and control, continuous learning, competence through knowledge and ability, nursing responsibility on an organisational level, application of skills, awareness of the individual’s needs and wholeness, mutual support, mutual relationships, collaborating on organisational and interpersonal levels, and exposure to challenges. This study explores RNs’ perceptions of their leadership linked to these themes, except the theme ‘exposure to challenges’, which was explored by Lillsjö et al. [2].

Altogether, there is an ongoing movement to more advanced care performed in municipal home healthcare from care performed in hospitals [4, 11–13]. Thus, there is an increased need for home healthcare, leading to increased demands for RNs [12]. RNs’ leadership close to older adults in home healthcare contributes to good and safe care for older adults [14, 38]. At the same time, RNs’ leadership is complex (1) and implies challenges (2). However, RNs’ leadership in municipal home healthcare, as well as their own perceptions of their leadership close to older adults has been sparsely explored.

### Aims

The aims are to explore and compare registered nurses’ perceptions of their leadership close to older adults in municipal home healthcare, as well as to correlate their perceptions with age and work experience.

## Method

### Design

The present study is part of a larger questionnaire survey exploring RNs’ (*n* = 71) professional competence in municipal home healthcare in Sweden. A non-experimental, and cross-sectional design was used [39]. Data was collected from May to November 2021. The statistics was performed using the Statistical Package for the Social Sciences (SPSS) version 28. The study was reviewed by the Health, Science, and Technology faculty’s review of research ethics at Karlstad University (Dnr. HNT 2020/618). The Strengthening the Reporting of Observational Studies in Epidemiology (STROBE) guidelines for cross-sectional study were used for reporting purposes [40, 41].

### Settings and participants

The target population was all RNs (*n* = 200) working as a leader close to older adults in home healthcare, in seven municipalities in two geographic areas in Sweden. RNs were included regardless of how many years they had worked as a RN. Exclusion criteria were RNs working as managers not providing direct care to older adults. The total number of participating RNs (*n* = 71) comprised 36% of the target population.

RNs in Swedish municipalities are awarded a licence after completing a three-year university education with a bachelor in science degree. In Sweden, there are several types of specialist education for RNs ranging from 60 to 90 European Credit Transfer System (ECTS) [42]. The most common specialist education among RNs in the Swedish municipalities is care for older followed by public health nurse [28].

### Questionnaire

The questionnaire in the larger study consisted of three sections: (1) Background variables, such as age, gender, work experience as a RN and specialist education; (2) Nurse Professional Competence Scale Short Form (NPC Scale-SF) with 35 questions [43]; and (3) 21 study-specific questions. Section three consisted of 21 questions about RNs’ leadership close to older adults in municipal home healthcare. Twenty of the questions in section three were created according to a systematic review of what RNs’ leadership implies close to older adults in municipal home healthcare [1]. The last question was modified from Andersson et al. [44] to suit the context of this study. The questions were developed in collaboration with a statistician and were discussed in a group of researchers with experience of home healthcare. The 18 questions included in this study, were derived from nine of Claesson et. al’s [1] themes of what RNs’ leadership implies close to older adults in municipal home healthcare: Trust and control (questions 1–3); continuous learning and competence through knowledge and ability (4–5); nursing responsibility on an organisational level (6); application of skills (7–9); awareness of the individual’s needs and wholeness (10–11); mutual support (12–13); mutual relationships (14–15) and collaborating on organisational and interpersonal levels (16–18). Cronbach’s alpha was .895 for these 18 questions. The 18 questions had response categories on an ordinal scale, with a scale range from “to a very low degree” (1), “to a low degree” (2), “to a quite low degree” (3), “neither a high or low degree” (4), “to a quite high degree” (5), “to a high degree” (6) to “to a very high degree” (7). The last three questions in section three were open-ended and the answers are reported elsewhere [2]. There was an opportunity for participants to add their own comments at the end of the questionnaire. In order to test the clarity and logic of the questions about leadership in section three, three RNs with experience in municipal care for older adults were interviewed. The test interviews elicited only a spelling mistake.

### Data collection and procedure

A convenience sample was used [39]. Data was collected from May to November 2021 in Sweden. Unit managers for RNs were contacted by e-mail after permission to do so was received from operation managers for municipal home healthcare. The unit managers were asked to send an e-mail to RNs with an invitation to participate. The e-mail included an information letter and a link to the web-based questionnaire. The information letter included information about the study’s aim, that participation was voluntary, that RNs could withdraw their participation without explanation, that data would be kept confidential, and that the RNs’ identity would be protected. The RNs gave their informed consent in the web-based questionnaire, and the questionnaire could only be answered with their informed consent. Two e-mail reminders were sent.

### Data analysis

The statistics was performed using the Statistical Package for the Social Sciences (SPSS) for Windows version 28. The background variables were presented via Mean (m), Standard Deviation (SD) and min-max [45]. RNs’ perceptions of their leadership were presented via Median (md), quartiles and min-max [45]. The internal losses of data were low and were not replaced or imputed [46].

The Mann-Whitney U test was used to test differences between two independent groups [45], RNs without and with specialist education. Spearman’s correlation coefficient (*r*_*s*_) [45] was used to measure correlations between all 18 questions and: age, work experience as a RN and work experience as a RN in home healthcare for older adults. For the statistical tests, a p value < .05 was considered statistically significant [45].

### Ethical considerations

Ethical principles in accordance with the Declaration of Helsinki [47] and Swedish ethic testing legalisation [48] were followed, with information to participants, informed consent and confidentiality. The results are described at a group level so that no individual RN can be identified.

## Results

The characteristics of RNs participating (*n* = 71) and across RNs without (*n* = 41) and with specialist education (*n* = 28) are presented in Table 1. Twenty-eight RNs reported to have a specialist education and *n* = 19 had a specialist education in care for older and public health nurse.

**Table 1:** Registered nurses’ (RNs) (*n* = 71) characteristics and across groups RNs without (*n =* 41) and with specialist education (*n* = 28)

Characteristics		RNs (*n* = 71)		RNs (*n* = 41) Without specialist education	RNs (*n* = 28) With specialist education
Female	*n* (%)	68 (96)			
Age	Mean (SD) min-max	48 (11.5) ^a^ 24–73	Mean (SD)	47.2 (13.1) ^a^	49.1 (8.9)
Year of nursing graduation	Median min-max	2006 ^a^ 1981–2020	Median	2008	2003 ^a^
Specialist education	*n* (%)	28 (41) ^b^			
Employment					
Permanent	*n* (%)	69 (97)			
Full time	*n* (%)	45 (63)			
Years worked					
As a RN	Mean (SD) min-max	16.2 (9.2) ^c^ 1–38	Mean (SD)	14.2 (9) ^e^	18.5 (8.7) ^b^
At current work place	Mean (SD) min-max	6.7 (6.5) ^c^ .3–35	Mean (SD)	6.2 (6.4) ^b^	6.4 (3.9) ^e^
As a RN caring for older adults	Mean (SD) min-max	11.8 (8.5) ^d^ .8–35	Mean (SD)	9.5 (8) ^f^	14.3 (7.4) ^f^
As a RN in home healthcare for older adults	Mean (SD) min-max	7.2 (6.3) ^c^ .4–29	Mean (SD)	4.9 (4.3) ^g^	10.5 (7.3) ^a^

^a^one missing, ^b^ two missing, ^c^ five missing, ^d^ twelve missing, ^e^ three missing, ^f^ six missing, ^g^ four missing

Table 2 presents a summary of RNs’ (*n* = 71) perceptions of their leadership close to older adults, as well as their perceptions of their leadership across RNs without (*n* = 41) and with (*n* = 28) specialist education.

**Table 2:** Registered nurses’ (RNs) (*n* = 71) perceptions of their leadership and across RNs without (*n* = 41) and with specialist education (*n* = 28)

Question	Variables^a^	RNs (*n* = 71)	RNs (*n* = 41) without specialist education		RNs (*n* = 28) with specialist education		
	Do you consider yourself as a leader close to the older adult	Median (quartiles^b^) min-max	Median (quartiles^b^) min-max	Mean rank	Median (quartiles^b^) min-max	Mean rank	Mann- Whitney U *p* value
1	…have leadership competence?	6 (5–7) 3–7	5 (5–6) 3–7	32.76	6 (5–7) 4–7	38.29	482 .242
2	…trust your competence to assess the older adults’ condition?	6 (5–7) 1–7	6 (5–7) 1–7	32.37	6 (5–7) 5–7	38.86	466 0.164
3	…trust care staffs’ competence to assess the older adults’ condition?	4 ^c^ (3–5) 2–7	4 ^c^ (3-5.5) 2–6	32.70	4.5 (3–5) 2–7	33.39	507 0.882
4	…have space in your work to develop sufficient competence in leadership?	4 ^d^ (4–5) 1–7	4 ^d^ (4–5) 1–7	37.27	4 (4–4) 2–7	29.45	673.5 0.085
5	…have access to competence development in leadership in a well-organised way in your work?	4 (3–4) 1–6	4 (3-4.5) 1–6	37.16	4 (3–4) 1–5	31.84	662.5 0.258
6	…have nursing responsibility on an organisational level, for example patient safety, creation of a safe and supportive work environment for care staff and evaluation of the care staff competence?	4 ^e^ (3–5) 1–7	4 ^e^ (3–5) 1–7	36.04	4 (3–5) 1–6	32.30	621.5 0.432
7	…that you can apply your professional experience in your work tasks?	6 (6–7) 4–7	6 (5–7) 4–7	30.15	7 (6–7) 5–7	42.11	375 0.009^*^
8	…that you are responsible for documentation of nursing interventions, according to current legislation?	6 ^e^ (5–7) 1–7	6 ^e^ (5–7) 1–7	33.60	6 (5.25-7) 4–7	35.79	524 0.636
9	…that you are responsible for evaluation of nursing interventions, according to current legislation?	6 ^e^ (5–7) 3–7	6 ^e^ (5–7) 3–7	34.64	6 (5–7) 3–7	34.30	565.5 0.943
10	…have the ability to assess the individual needs of the older adult?	6 (6–7) 4–7	6 (5.5–6.5) 4–7	30.48	7 (6–7) 4–7	41.63	388.5 0.014*
11	…have the ability to assess the older adults’ needs based on a holistic view?	6 ^e^ (5.75-7) 4–7	6 (5-6.5) 4–7	29.71	7 ^e^ (6–7) 5–7	41.78	357 0.009*
12	…that you support colleagues i.e. registered nurses?	6 (6–7) 4–7	6 (6–7) 4–7	31.70	7 (6–7) 5–7	39.84	438.5 0.071
13	…get support from colleagues i.e. registered nurses?	6 (6–7) 3–7	6 (6–7) 3–7	32.29	6.5 (6–7) 4–7	38.96	463 0.147
14	…create good relationships with the older adult, through support, understanding and encouragement?	6 (6–7) 4–7	6 (6–7) 4–7	31.56	7 (6–7) 5–7	40.04	433 0.057
15	…create good relationships with the older adults’ next of kin, through support, understanding and encouragement?	6 ^e^ (6–7) 4–7	6 ^e^ (5.25-7) 4–7	30.55	7 (6–7) 5–7	40.14	402 0.035*
16	…collaborate on an organisational level, for example with your operation manager?	5 (4–6) 1–7	5 (4–6) 2–7	34.65	6 (4–6) 1–7	35.52	559.5 0.856
17	…collaborate on an interpersonal level, i.e. interaction with the older adult?	6 ^d^ (6–7) 4–7	6 ^e^ (5-6.75) 4–7	28.24	7 ^e^ (6–7) 5–7	42.54	309.5 0.002*
18	…collaborate on an interpersonal level, i.e. interaction with next of kin to the older adult?	6 ^e^ (5–7) 4–7	6 ^e^ (5-6.75) 4–7	29.38	6.5 (6–7) 4–7	41.82	355 0.007*

^a^Scale range 1 = to a very low degree, 2 = to a low degree, 3 = to a quite low degree, 4 = neither a high or low degree, 5 = to a quite high degree, 6 = to a high degree and 7 = to a very high degree

^b^The 25th and 75th percentiles

^c^Four missing, ^d^ Two missing, ^e^ One missing

* Statistically significant *p* < .05

### Comparison between registered nurses without and with specialist education

There were statistically significant differences between RNs’ perceptions of their leadership if they had specialist education or not (Table 2). RNs with specialist education perceived to a higher degree that they could apply their professional experience in their work tasks (*p* = .009) (Question 7); had the ability to assess individuals’ needs (*p* = .014) (Question 10) and needs based on a holistic view of the older adult (*p* = .009) (Question 11). RNs with specialist education perceived to a higher degree that they created good relationships with the older adults’ next of kin, through support, understanding and encouragement (*p* = .035) (Question 15); collaborated on an interpersonal level, i.e. interaction with the older adult (*p* = .002) (Question 17); and collaborated on an interpersonal level, i.e. interaction with next of kin to the older adult (*p* = .007) (Question 18).

### Correlation between registered nurses’ perceptions of their leadership with age and work experiences

There was a statistically significant positive correlation between RNs’ age (Table 3) and whether they perceived themselves to have competence in leadership (*r*_*s*_ = .24; *p* = .049) (Question 1); whether they trusted care staffs’ competence to assess the older adults’ condition (*r*_*s*_ = .39, *p* = .001) (Question 3); and whether they applied their professional experience in their work tasks (*r* _*s*_* = * .32; *p* = .006) (Question 7).

**Table 3:** Spearman’s correlation coefficient (*r*_*s*_) between registered nurses’ (RNs’) perception of their leadership and RNs’ age, work experience as a RN and work experience as a RN in home healthcare for older adults

Question	Variables	Age	Work experience as a RN	Work experience as a RN in home healthcare for older adults
	Do you consider yourself as a leader close to the older adult	Spearman’s correlation coefficient (*r*_*s*_)	Spearman’s correlation coefficient (*r*_*s*_)	Spearman’s correlation coefficient (*r*_*s*_)
1	…have leadership competence?	.24 *^a^	.41 **^b^	.29 *^b^
2	…trust your competence to assess the older adults’ condition?	0.12 ^a^	0.31 *^b^	0.23 ^b^
3	…trust care staffs’ competence to assess the older adults’ condition?	0.39 **^b^	0.22 ^e^	0.21 ^e^
4	…have space in your work to develop sufficient competence in leadership?	0.13 ^c^	0.16 ^f^	0.06 ^f^
5	…have access to competence development in leadership in a well-organised way in your work?	0.14 ^a^	0.03 ^b^	0.21 ^b^
6	…have nursing responsibility on an organisational level, for example patient safety, creation of a safe and supportive work environment for care staff and evaluation of the care staff competence?	0.04 ^d^	− 0.09 ^g^	0.22 ^g^
7	…that you can apply your professional experience in your work tasks?	0.32 **^a^	0.26 *^b^	0.27 *^b^
8	…that you are responsible for documentation of nursing interventions, according to current legislation?	0.19 ^d^	0.18 ^g^	0.15 ^g^
9	…that you are responsible for evaluation of nursing interventions, according to current legislation?	0.06 ^d^	0.16 ^g^	0.18 ^g^
10	…have the ability to assess the individual needs of the older adult?	0.07 ^a^	0.17 ^b^	0.25 *^b^
11	…have the ability to assess the older adults’ needs based on a holistic view?	0.10 ^d^	0.20 ^g^	0.33 **^g^
12	…that you support colleagues, i.e. registered nurses?	− 0.07 ^a^	0.12 ^b^	0.21 ^b^
13	…get support from colleagues, i.e. registered nurses?	0.04 ^a^	0.11 ^b^	0.29 *^b^
14	…create good relationships with the older adult, through support, understanding and encouragement?	− 0.05 ^a^	0.01 ^b^	0.26 *^b^
15	…create good relationships with the older adults’ next of kin, through support, understanding and encouragement?	− 0.13 ^d^	0.05 ^g^	0.20 ^g^
16	…collaborate on an organisational level, for example with your operation manager?	− 0.13 ^a^	− 0.02 ^b^	0.06 ^b^
17	…collaborate on an interpersonal level, i.e. interaction with the older adult?	− 0.05 ^c^	0.14 ^f^	0.14 ^f^
18	…collaborate on an interpersonal level, i.e. interaction with next of kin to the older adult?	− 0.07 ^d^	0.13 ^g^	0.13 ^g^

*Correlation is significant at a level of *p* < .05 ** Correlation is significant at a level of *p* < .01

^a^*n* = 70, ^b^*n* = 66, ^c^*n* = 68, ^d^*n* = 69, ^e^*n* = 63, ^f^*n* = 64, ^g^*n* = 65

There was a statistically significant positive correlation between RNs’ work experience as a RN (Table 3) and whether they perceived to have competence for their leadership (*r*_*s*_ = .41; *p* < .001) (Question 1); whether they trusted their competence to assess the older adults’ condition (*r*_*s*_ = .31; *p* = .011) (Question 2); and whether RNs perceived that they were able to apply their professional experience in their work (*r*_*s*_* = *.26; *p* = .033) (Question 7).

RNs’ work experience as a RN in home healthcare for older adults was statistically significantly positively correlated (Table 3) with their perceptions of: whether they had leadership competence (*r*_*s*_ = .29; *p* = .017) (Question 1); whether they applied their professional experience in their work (*r* _*s*_* = *.27; *p* = .027) (Question 7); and whether they had the ability to assess the individual needs of the older adult (*r*_*s*_ = .25; *p* = .04) (Question 10) and needs based on a holistic view (*r*_*s*_ = .33; *p* = .006) (Question 11). Moreover, statistically significant positive correlations were found between RNs’ work experience as a RN in home healthcare for older adults and whether RNs perceived that they received support from colleagues i.e. RNs (*r* _*s*_* = *.29; *p* = .017) (Question 13) and whether they created good relationships with the older adults through support, understanding and encouragement (*r* _*s*_ = .26; *p* = .037) (Question 14).

## Discussion

The results showed RNs’ perceptions of their leadership close to older adults in municipal home healthcare. Overall, RNs perceived their ability as leaders close to older adults as high. However, RNs had neither low or high trust in care staffs’ competence to assess the older adults’ condition; having space in their work to develop sufficient competence in leadership; having access to competence development in leadership in a well-organised way at work; and having nursing responsibility on an organisational level.

RNs had neither low or high trust in care staffs’ competence to assess the older adult’s condition. Earlier research describes how RNs in home healthcare often have to rely on care staffs’ competence and judgement. Care staff are often the ones meeting the older adults most regularly, and they communicate changes in the older adults’ condition to RNs [22, 23]. The high number of older adults within RNs’ responsibility, implies that RNs have to delegate home healthcare tasks to care staff [20, 24]. This means that the organisation relies on care staff having the competence to identify changes in the older adults’ condition, such as emergency symptoms [49]. In light of this and the results of this study, there seems to be a difference between how home healthcare is organised and RNs’ perceptions of their trust in care staffs’ competence. Earlier research shows that RNs perceive that care staffs’ low competence affects the older adults care and creates suffering for the older adult [2]. According to Kouzes and Posner [34], mutual trust is needed between leaders and followers for a functioning leadership, trust facilitates collaboration and that the team work towards common goals. Due to this, further research is needed on RNs’ experiences of their leadership for care staff in home healthcare.

Furthermore, this study revealed that RNs perceived neither in a low or high degree that they had nursing responsibilities on an organisational level, for example patient safety, creation of a safe and supportive work environment for care staff and evaluation of the care staff competence. Lindberg et al. [16] on the other hand, showed that RNs in home healthcare adapt to organisational preconditions, such as taking responsibility for training and supporting care staff with limited knowledge and competence, to promote safe home healthcare for older adults. Today in Swedish municipalities, there is a lack of care staff with education in care and social care [19]. Care staff in home healthcare lack support, with the result that they have to make their own decisions based on limited information [50], and their responsibilities in healthcare tasks are unclear [51]. Moreover, care staff in the municipalities have concerns about contacting RNs due to fear of not being believed or calling RNs too often [52]. The Health and Social Care Inspectorate in Sweden has highlighted the importance of care staff having support from RNs in the municipality to promote safe care [53]. Therefore, the results of this study, indicate that more knowledge is needed about RNs’ experiences of their leadership on an organisational level in home healthcare, such as experiences concerning patient safety and supporting care staff, as well as care staffs’ experiences of RNs’ leadership in home healthcare.

RNs in this study perceived neither in a low or high degree that they have space in their work to develop sufficient competence in leadership, and access to competence development in leadership in a well-organised way at work. Research shows that RNs want leadership development [2, 54]. Cummings et al. [35] showed that leadership development interventions have positive outcomes for leadership in nursing. Due to this, enabling RNs in home healthcare to regularly develop their competence in leadership, may be a way to strengthen RNs’ leadership. However, for RNs to be able to participate in professional development, there is a need for organisational preconditions supporting professional development [55, 56]. In this study, RNs perceived neither in a low or high degree that they have space and access to competence development in leadership. Funding and time to participate in professional development were described as organisational hindrances [56]. Lack of time in home healthcare was reported [57, 58]. Therefore, there is a need for organisational preconditions to enable RNs in home healthcare to develop their competence in leadership.

The results showed statistically significant differences in RNs’ perceptions of their leadership close to older adults, between RNs without and with specialist education. The meaning of being a specialist nurse differs internationally, for example in education level [59]. However, the results of this study indicated that specialist education may promote strengthened leadership of RNs. Earlier research has shown similar results. Green [60] showed that RNs undergoing a one-year specialist education in district nursing felt that they developed their leadership ability. Furthermore, RNs with specialist education reported a higher self-rated satisfaction with their own professional competence, compared to RNs without specialist education [27]. Berthelsen et al. [61] showed that RNs undergoing specialist education in community and primary healthcare perceived that they strengthened their knowledge in collaboration and cooperation with other healthcare professionals. In light of this, and the results of this study, specialist education may strengthen RNs’ leadership. Accordingly, increased knowledge is needed of how specialist education could facilitate RNs’ leadership. This can be used to develop specialist educations for optimal outcomes. If RNs in home healthcare are given the opportunity by their employer to undergo a specialist education, this could also encourage RNs to remain in home healthcare. This may contribute to reducing the challenges of recruiting RNs to home healthcare [28].

The results pointed out that RNs with specialist education perceived to a higher degree than those without specialist education, that they had the ability to assess the individual needs of older adults, as well as needs based on a holistic view. In earlier research, the ability to see older adults’ individual needs through adopting a holistic view, not only focusing on physical shortcomings, has been perceived as contributing to safe home healthcare [62]. Moreover, the result of this study showed that RNs with specialist education perceived to a higher degree that they collaborated on an interpersonal level, i.e. interacted with older adults and next of kin, and created good relationships with the older adults’ next of kin. This could be seen in line with person-centred care. Person-centred care can be described as an approach where the person’s view of their life situation and conditions is at the centre of the care. The patient and next of kin are active partners in the care and in decision-making [63, 64]. The International Council of Nurses [65] states that RNs should work towards person-centred care. RNs’ leadership close to older adults in home healthcare can contribute to older adults being active partners in their care [38]. The results of this study indicate that specialist education may facilitate RNs leading for a person-centred care. However, to enable a person-centred care [66] and to favour the older adult’s individual goals [67], there must also be organisational preconditions [66, 67]. Therefore, a deeper understanding is needed of factors that facilitate person-centred care for older adults in home healthcare, both educational and organisational factors.

The results of this study, in general, showed no strong correlation coefficients between RNs’ perceptions of their leadership and age and work experience. Earlier research has shown equivocal results of associations between leadership and age and experience [35]. At the same time, newly graduated RNs have reported that their leadership is a continuous growth process that develops over time [68]. Therefore, more research is needed. However, this study showed the strongest correlation between RNs’ work experience as an RN and their perception of their own leadership competence. Moreover, the results showed statistically significant correlations between RNs’ perception of their leadership and experience as an RN in home healthcare for older adults. This, highlight the value of retain RNs with work experience in home healthcare. Clinical experience in addition to formal education, is required for RNs’ development from novice to expert [69]. However, clinical experience is not only about the passage of time but also about understanding situations through practical situations, and taking appropriate actions based on this [69]. Thus, increased knowledge of how work experience may affect RNs’ leadership is of value.

### Limitations

The participants were invited to participate through their unit managers via e-mail, which may be a limitation since not all RNs may have received an invitation. The response rate was 36%. This could lead to a sampling bias that affects the representativeness of the overall population [70] and may reduce internal validity [39]. Questionnaires where the participants only answer questions determined by the researcher may be a limitation [39]. However, this is a limitation for most questionnaires and the participants had the opportunity to add comments at the end of the questionnaire. The questions in this study were derived from a systematic review [1], of what RNs’ leadership implies close to older adults in municipal home healthcare. This could be seen as a strength. Another strength is that RNs with experience in municipal care for older adults were interviewed to test the clarity and logic of the questions. The questions were also discussed by a group of researchers with experience of home healthcare. Cronbach’s alpha was .895 for the questions in this study. No further analyses were conducted of the questions, which is recommended if the questions are used again to strengthen reliability. The results should be read with the understanding that it might be a risk for mass-significance to conduct many statistical tests simultaneously. Due to non-random sample the results cannot be generalised. This reduces external validity. However, the result may reflect other RNs’ perceptions of their leadership when working in similar contexts. The results could be valuable and relevant to clinical practice and education, as well as for further research.

## Conclusions

Specialist education for RNs may strengthen RNs’ leadership in home healthcare for older adults and may facilitate person-centred care. Further research is needed of RNs’ experiences of their leadership to gain new knowledge of RNs’ leadership in home healthcare, as well as care staff’s experiences of RNs leadership in municipal home healthcare.

## Data Availability

The datasets generated and analysed during the current study are not publicly available due to privacy and ethical restrictions but are available from the corresponding author upon reasonable request.
